# Self-Managed Abortion Attempts Before vs After Changes in Federal Abortion Protections in the US

**DOI:** 10.1001/jamanetworkopen.2024.24310

**Published:** 2024-07-30

**Authors:** Lauren Ralph, Rosalyn Schroeder, Shelly Kaller, Daniel Grossman, M. Antonia Biggs

**Affiliations:** 1 Advancing New Standards in Reproductive Health, Bixby Center for Global Reproductive Health, Department of Obstetrics, Gynecology and Reproductive Sciences, University of California, San Francisco, Oakland

## Abstract

**Importance:**

With decreasing access to facility-based abortion in the US, an increase in self-managed abortion (SMA) using various methods is anticipated. To date, no studies have examined changes in SMA in the shifting policy landscape.

**Objective:**

To estimate changes in SMA prevalence among the general US population from before to after the Supreme Court’s June 2022 decision overturning federal abortion protections.

**Design, Setting, and Participants:**

Serial cross-sectional surveys were administered throughout the US from December 10, 2021, to January 11, 2022, and June 14 to July 7, 2023. Participants included online panel members assigned female sex at birth, ages 18 to 49 years (or ages 15-17 years if a household member), who were English- or Spanish-speaking.

**Exposure:**

Year of the survey (2021-2022 vs 2023).

**Main Outcome and Measures:**

Participants were asked whether they had “ever taken or done something on their own, without medical assistance, to try to end a pregnancy” and, if so, details of their experience. Changes in the weighted SMA prevalence between survey years were examined, factors associated with SMA experience were identified, and projected lifetime SMA prevalence was calculated using discrete-time event history models, accounting for abortion underreporting.

**Results:**

Median age of the participants was 32.5 (IQR, 25-41) years in 2021-2022 (n = 7016) and 32.0 (IQR, 24-40) in 2023 (n = 7148). Across both years, approximately 14% were non-Hispanic Black, 21% were Hispanic, and 54% were non-Hispanic White. The weighted adjusted proportion that ever attempted SMA was 2.4% (95% CI, 1.9%-3.0%) in 2021-2022 and 3.4% (95% CI, 2.8%-4.0%) in 2023—an increase of 1.0% (95% CI, 0.2%-1.7%; *P* = .03). Projected lifetime SMA prevalence accounting for abortion underreporting was 10.7% (95% CI, 8.6%-12.8%). In adjusted analyses, SMA experience was higher among non-Hispanic Black (4.3%; 95% CI, 2.8%-5.8%) vs other racial and ethnic (2.7%; 95% CI, 2.2%-3.1%) groups (*P* = .04) and sexual and gender minority (5.0%; 95% CI, 3.4%-6.6%) vs heterosexual or cisgender (2.5%; 95% CI, 2.0%-2.9%) participants (*P* < .001). Approximately 4 in 10 (45.3% in 2021 and 39.0% in 2023) SMA attempts occurred before age 20 years. The methods used included herbs (29.8% [2021-2022] vs 25.9% [2023]), physical methods (28.6% [2021-2022] vs 29.7% [2023]), or alcohol or other substances (17.9% [2021-2022] vs 18.6% [2023]). Few participants (7.1% [2021-2022] vs 4.7% [2023]) sought emergency care for a complication.

**Conclusions and Relevance:**

In this serial nationally representative survey study, increased SMA was observed following the loss of federal abortion protections. The findings revealed increased SMA use among marginalized groups, most often with ineffective methods. These findings suggest the need to expand access to alternative models of safe and effective abortion care and ensure those seeking health care post-SMA do not face legal risks.

## Key Points

**Question:**

Did the prevalence of self-managed abortion increase following the Supreme Court’s June 2022 decision overturning federal protections on abortion?

**Findings:**

In this cross-sectional survey study, with surveys administered to different cohorts in December 2021 and January 2022 (n = 7016) and June and July 2023 (n = 7148), the proportion of the US female population of reproductive age reporting having ever self-managed an abortion increased from 2.4% to 3.3%. The projected lifetime experience with self-managed abortion, adjusted for underreporting of abortion, was 10.1%.

**Meaning:**

These findings suggest an increased prevalence of self-managed abortion in the US; self-managed abortion should continue to be monitored carefully as barriers to facility-based care broaden.

## Teaser

This survey study examines changes in self-managed abortion attempts from before to after the 2022 Supreme Court decision to overturn federal protections on abortion.

## Introduction

With the Supreme Court’s decision in *Dobbs vs Jackson Women’s Health Organization* in June 2022 overturning federal protections on abortion, the landscape of abortion access in the US has changed substantially. As of June 2024, 21 states had banned or severely restricted access to abortion.^1^ Emerging evidence indicates that travel for abortion to states where it remains legally protected has surged.^2^ However, for people who are pregnant and unable to travel, an increase in unwanted births^3,4^ and in abortion occurring outside of the formal health care system, also known as self-managed abortion (SMA),^5^ is expected.

Self-managed abortion typically includes any action taken to end a pregnancy (confirmed or suspected) without medical supervision and includes self-sourcing the World Health Organization–recommended medications (ie, mifepristone and misoprostol); ingesting herbs, alcohol, or other substances; or using physical methods such as punching oneself in the stomach.^6,7^ Before the *Dobbs* ruling, numerous studies highlighted experience with SMA among people in the US, with estimates ranging from 2% to 7% of those seeking abortion care^8,9,10,11^ to 28% of those searching online for information about abortion,^12^ and increased among those facing barriers to facility-based abortion care.^9,12^ Emerging evidence indicates that one type of SMA, self-sourcing medication abortion pills, has increased since the *Dobbs* decision, with requests to one online telemedicine service more than doubling since June 2022.^13^

To fully document the impact outcome of the *Dobbs* decision on access to abortion, population-based studies examining prevalence of SMA are needed. A 2017 study found that 2% of the US female population of reproductive age reported having ever attempted SMA.^14^ In the present study, we used a similar method and serial cross-sectional surveys with nationally representative samples of the US population to examine changes in SMA prevalence from just over 1 year before and 1 year after the *Dobbs* decision, as well as describe people’s experiences with SMA. Even with underreporting of abortions in self-reported surveys,^15^ this study can provide evidence of trends in self-managed abortion in an increasingly restricted landscape.

## Methods

This survey study relied on data from surveys administered in December 10, 2021, to January 11, 2022, and June 14 to July 7, 2023, by a public opinion and market research firm to members of its online panel. Panel members are recruited from a probability sample of US addresses to be representative of the adult noninstitutionalized population. Study participants received compensation through the firm’s points program at values equivalent to $4 to $6. Participants and the parents of minor adolescents provided electronic informed consent. All study activities were approved by the University of California San Francisco Institutional Review Board. This study followed the Strengthening the Reporting of Observational Studies in Epidemiology (STROBE) reporting guideline.^16^

For both surveys, panel members aged 18 to 49 years whose sex assigned at birth was female received an email invitation from the research firm to complete an online survey on reproductive health experiences and opinions. In addition, panel members with female household members aged 15 to 17 years received an email asking them to invite that adolescent. Invited participants received reminders at 3 and 7 days after the initial invitation, and data collection closed when sample size targets (n = 7000) were met. Data collection was conducted for 32 days in 2021-2022 and 23 days in 2023.

### Measures

The primary outcome of interest was experience with SMA, assessed using a series of 2 questions. The first, from prior research,^14^ asked participants whether they had “ever taken or done something on [their] own, without medical assistance, to try to end a pregnancy,” with “yes,” “no,” or “I don’t know” response options. The second, displayed only to those who selected no or skipped the first question, presented a list of SMA methods and asked participants to select any they had ever used “to try to end a pregnancy on [their] own, without medical assistance.” This 2-step question format was chosen based on research reporting higher recall of SMA experiences with this approach.^12^ A longer description of SMA, developed using cognitive interviews,^17^ preceded the first question.

Participants who responded affirmatively to either question were asked follow-up questions including the method used; the number of SMA attempts; their age at the first and, if applicable, the most recent attempt; pregnancy testing before the SMA attempt (yes, no, or I don’t remember) and result (positive, negative, or unclear); reason they suspected pregnancy (positive pregnancy test, missed period, condom broke, missed pills or birth control, sex without birth control, and pregnancy symptoms); where (state or country) they lived at the most recent attempt; whether the methods worked to end the pregnancy (yes, no, or not sure); what they did about the pregnancy if the methods did not work (went to a hospital or clinic for abortion, continued with the pregnancy, had a miscarriage later in pregnancy, or nothing); whether they experienced a medical complication, defined as “something that required treatment by a doctor or nurse”; complication type (bleeding, pain, fever, nausea, vomiting, and diarrhea [2023 only]); and whether they sought follow-up care at a “hospital, emergency room, or urgent care.” A final question asked participants their reasons for SMA. Questions were mostly close ended; for methods used, reasons for suspecting pregnancy, what they did about the pregnancy, complications, and reasons for SMA, participants could also write responses. For methods used, reason for suspecting pregnancy, complications, and reasons for SMA, participants could select multiple response options.

We used variables regularly collected on all adult panel members for participants’ self-reported age; race and ethnicity (assessed separately at collection but combined for analysis); state of residence; household income and size; highest level of educational attainment; marital status; and lesbian, gay, bisexual, transgender, queer, or nonbinary/gender nonconforming (LGBTQ) identity. We calculated the percent federal poverty level using the most recent thresholds available.^18^ We surveyed all participants on nativity and their perception of their family’s socioeconomic status during their adolescence. For minors, we used parents’ report of household income and size and state of residence; however, we relied on minors’ self-reports of their race and ethnicity. Survey questions also assessed lifetime and past-year abortion experience.

### Statistical Analysis

When noted, survey weights generated by the research firm were applied to align the sample with benchmarks on race and ethnicity, age, educational level, census region, and metropolitan status from the most recent Current Population Survey.^19^ We used weighted descriptive statistics to identify participant sociodemographic characteristics and generate estimates of the proportion who had ever attempted SMA each year. For all analyses, we removed SMA attempts that occurred outside the US and those that used emergency contraception before confirming pregnancy as their only method, since this may represent appropriate use of emergency contraception and not abortion. We conducted sensitivity analyses. The first removed attempts where taking a hot bath or shower was their only method, since this method may be more likely to be misreported, and the second was removing attempts where the pregnancy was not confirmed first. A confirmed pregnancy was defined as a positive pregnancy test, the pregnancy ending in an in-clinic abortion or birth, or a write-in response indicating pregnancy confirmation.

To evaluate whether the proportion who attempted SMA changed between 2021 and 2023, we constructed a weighted logistic regression model with SMA as the dependent variable and survey year as the independent variable; change over time was significant if *P* < .05 on year. In adjusted analyses, we added covariates that are time-invariant (race and ethnicity, place of birth, and sexual or gender identity) or likely preceded the SMA attempt (family socioeconomic situation during adolescence) to ensure temporal associations. We included race and ethnicity as a proxy for experiences of medical mistreatment or racism, which may lead to a need or preference for accessing abortion outside the formal health care system. Using this same modeling approach but restricted to the 2023 data, we then examined sociodemographic factors associated with having ever attempted SMA. For ease of interpretation, we present marginal predicted probabilities of SMA experience. To avoid centering whiteness or other socially advantaged groups, we compared the likelihood of SMA within a given subgroup with everyone not in that subgroup vs an arbitrarily selected reference group, such as White race. To examine whether SMA attempts changed between 2021 and 2023 within subgroups, we ran weighted, adjusted logistic regression models; change over time within the subgroup was considered significant if *P* < .05 on an interaction term of year × subgroup. For these models, we account for clustering of observations among participants that participated in both the 2021 and 2023 surveys (n = 3400). Postestimation margins commands were used to estimate predicted proportions within subgroups from adjusted models.

Among participants who reported having attempted SMA, we used unweighted descriptive statistics to describe details of their experience. Year and decade of SMA attempts were calculated using the participant’s current age, age at SMA attempt, and survey year. We projected lifetime experience with SMA from our cross-sectional data using discrete-time event models analyzed with the Stata TFR2 package.^20^ This approach uses the participant’s age at first SMA, current age, and survey year to calculate age-specific rates and then combines those to arrive at a lifetime fertility (herein, SMA) rate.

Underreporting of abortion is a common challenge in survey research.^21,22^ To adjust for underreporting, we developed a multiplier by comparing the proportion of the 2021 sample reporting a past-year abortion with the proportion of the US female population that had an abortion in 2020 (1.32%/0.62% = 2.1); estimates were derived from the Guttmacher 2020 Abortion Provider Census^23^ and census estimates of the 2020 female population aged 18 to 49 years.^24^ Arithmetic to calculate the multiplier is provided in the eMethods in Supplement 1. We applied this multiplier to SMA prevalence estimates in sensitivity analyses.

All analyses were conducted using Stata, version 15 (StataCorp LLC) statistical software. A priori calculations indicated 80% power to detect a half (0.5%) percentage point change in SMA prevalence with a sample size of 6442 per year. Two-sided, unpaired testing was conducted.

## Results

In 2021, 7388 of 15345 adult female panel members invited (48%) were screened for eligibility. Of 7360 eligible individuals, 6841 (93%) completed the survey. An additional 175 adolescents aged 15 to 17 years (of 358 eligible [48%]) completed the survey. In 2023, 7286 of 13072 (56%) adult female panel members invited were screened for eligibility. Of the 7094 eligible individuals, 6785 (96%) completed the survey. An additional 363 adolescents aged 15 to 17 years (of 406 eligible [89%]) completed the survey. Thus, there were 7016 participants in 2021-2022 and 7148 in 2023.

Mean age of study participants was 32.5 (IQR, 25-41) years in 2021 and 32.0 (IQR, 24-40) years in 2023; across both years, approximately 14% were non-Hispanic Black, 21% were Hispanic, and 54% were non-Hispanic White (Table 1). With respect to age, race and ethnicity, and level of education, the study samples were comparable to census estimates (eTable in Supplement 1). Less than 1% (0.62% in 2021 and 0.88% in 2023) of the participants reported a past year abortion (Table 1). Comparing national estimates of past-year abortion (1.32%) with estimates from the survey, we estimate underreporting of abortions of 47%, resulting in a multiplier of 2.1 (eMethods in Supplement 1).

**Table 1.** Sociodemographic and Reproductive Characteristics of the 2021-2022 and 2023 Study Populations

Characteristic	Unweighted No. (weighted %)^a^	
	December 2021-January 2022 (n = 7016)	June-July 2023 (n = 7148)
Age, y		
15-17	175 (9.0)	363 (8.9)
18-24	439 (15.7)	536 (19.0)
25-29	1025 (15.6)	881 (14.5)
30-39	2684 (31.4)	2556 (30.2)
40-49	2693 (28.5)	2812 (27.4)
Race and ethnicity^b^		
Hispanic	1397 (21.3)	1435 (21.5)
Non-Hispanic Asian or Pacific Islander	248 (5.7)	274 (5.1)
Non-Hispanic Black	598 (13.6)	609 (13.9)
Non-Hispanic White	4530 (54.9)	4537 (54.6)
Non-Hispanic Multiple or Other	242 (4.6)^c^	293 (5.4)^d^
Household income, % federal poverty level		
<100	1123 (12.2)	1050 (10.3)
100-199	1117 (14.1)	1045 (13.0)
≥200	4776 (73.8)	5053 (76.7)
Educational attainment^e^		
<HS diploma	262 (8.2)	296 (8.3)
HS diploma	895 (21.6)	962 (24.1)
Some college	1893 (29.3)	1951 (28.2)
≥Bachelor’s degree	3791 (40.9)	3576 (39.5)
Family’s financial situation during adolescence	n = 6841	n = 6785
Pretty well off financially	1238 (18.7)	1117 (15.6)
About average	4146 (59.2)	4202 (58.8)
Poor	1594 (21.5)	1779 (24.9)
Refused	38 (0.7)	50 (0.7)
Marital status		
Currently married	3670 (54.1)	3530 (47.6)
Widowed/divorced/separated	737 (7.4)	653 (6.1)
Never married	2609 (38.5)	2602 (46.3)
Place of birth		
US	6081 (85.3)	6216 (87.0)
Outside the US	935 (14.7)	909 (12.7)
Refused	0	23 (0.3)
Current geographic region of residence		
Northeast	1090 (16.9)	1044 (16.9)
Midwest	1769 (20.2)	1829 (20.2)
South	2509 (38.7)	2564 (38.6)
West	1648 (24.3)	1711 (24.3)
LGBTQ identity		
No	6115 (86.4)	6027 (82.2)
Yes	897 (13.5)	1117 (17.8)
Refused/missing	4 (<0.1)	4 (<0.1)
Lifetime abortion		
Yes	813 (10.7)	849 (10.8)
No	6185 (88.9)	6262 (88.8)
Refused	18 (0.4)	37 (0.4)
Past year abortion		
Yes	35 (0.62)	60 (0.88)
No	6802 (99.4)	6724 (99.1)
Refused	4 (0.02)	1 (0.01)

Abbreviations: HS, high school; LGBTQ, lesbian, gay, bisexual, transgender, queer, or nonbinary/gender nonconforming.

^a^ All values are weighted column percentages. Weights generate estimates representative of the US noninstitutionalized female population ages 15 to 49 years with respect to census age, race and ethnicity, region of residence, educational level, household income, and language.

^b^ Race and ethnicity were self-reported by participants to the market research firm (adults) and in the UCSF survey (teens). We collapsed responses to 5-part combined race and ethnicity variable.

^c^ Includes 21 American Indian or Alaska Native only, 1 Native Hawaiian Pacific Islander only, and 220 reporting multiple races.

^d^ Includes 25 American Indian or Alaska Native only and 268 reporting multiple races.

^e^ Only among individuals older than 17 years.

The unadjusted weighted proportion of the sample that reported having ever attempted SMA was 2.4% (95% CI, 1.9%-3.0%) in 2021 and 3.4% (95% CI, 2.8%-4.0%) in 2023, which was a significant increase of 1.0% (95% CI, 0.2%-1.7%; *P* = .01). Restricted to cases of a confirmed pregnancy, the proportion that attempted SMA was 1.4% (95% CI, 1.0%-1.9%) in 2021 and 1.8% (95% CI, 1.4%-2.3%) in 2023; this change was not significant (*P* = .16). Accounting for estimated underreporting of abortion, the proportion that had ever attempted SMA overall was 5.0% (95% CI, 4.0%-6.3%) in 2021 and 7.1% (5.9%-8.4%) in 2023. Using discrete-time models with the 2023 data, the projected lifetime prevalence of SMA was 5.1% (95% CI, 4.1%-6.1%); accounting for underreporting of abortion, this figure was 10.7% (95% CI, 8.6%-12.8%) (Table 2).

**Table 2.** Unadjusted and Weighted Estimates of the Prevalence of SMA Attempts From 2021 to 2023, Overall and Accounting for Underreporting of Abortion

Variable	Dec 2021-Jan 2022 (n = 7016)		June-July 2023 (n = 7148)		Absolute difference, 2021-2023, % (95% CI)	*P* value^a^
	No.	Weighted % (95% CI)	No.	Weighted % (95% CI)		
Ever attempted SMA	168	2.4 (1.9-3.0)	236	3.4 (2.8-4.0)	1.0 (0.2-1.7)	.01
Accounting for estimated underreporting of abortion^b^	NA	5.0 (4.0-6.3)	NA	7.1 (5.9-8.4)	NA	NA
Ever attempted SMA, removing hot baths	157	2.2 (1.8-2.8)	228	3.3 (2.7-4.0)	1.1 (0.4-1.8)	.005
Accounting for estimated under-reporting of abortion^b^	NA	4.6 (3.8-5.9)	NA	6.9 (5.7-8.4)	NA	NA
Ever attempted SMA in a confirmed pregnancy^c^	89	1.4 (1.0-1.9)	131	1.8 (1.4-2.3)	0.4 (−0.1-1.0)	.16
Accounting for estimated underreporting of abortion^b^	NA	2.9 (2.1-4.0)	NA	3.8 (2.9-4.8)	NA	NA
Lifetime prevalence of SMA^d^	NA	NA	NA	5.1 (4.1-6.1)	NA	NA
Accounting for estimated underreporting of abortion^b^	NA	NA	NA	10.7 (8.6-12.8)	NA	NA

Abbreviations: NA, not applicable; SMA, self-managed abortion.

^a^ *P* value on difference in the prevalence of SMA by year of the survey from a weighted logistic regression model, with year as the only independent variable.

^b^ Estimate of underreporting of abortion was 47%, necessitating multiplying estimates by 2.1. Details on the calculation of this multiplier are in the eMethods in Supplement 1.

^c^ Confirmed pregnancy defined as reporting a positive pregnancy test before SMA or the pregnancy outcome being a facility-based abortion or continuing the pregnancy.

^d^ Lifetime prevalence of SMA estimated using discrete-time event models.

In multivariable analyses, the adjusted weighted proportion of the sample that reported having ever attempted SMA was 2.4% (95% CI, 1.9%-3.0%) in 2021 and 3.3% (95% CI, 2.7%-3.9%) in 2023, and the increase in SMA attempts over time remained significant (*P* = .03). In 2023, the adjusted proportion attempting SMA was higher among non-Hispanic Black individuals (5.1%; 95% CI, 2.9%-7.4%) vs all other race and ethnicity groups (3.1%; 95% CI, 2.5%-3.7%) (*P* = .04). Conversely, the prevalence of SMA was lower among non-Hispanic White individuals (2.7%; 95% CI, 2.0%-3.4%) vs all other groups (4.0%; 95% CI, 2.9%-5.2%) (*P* = .05) and non-Hispanic Asian and Pacific Islander (0.7%; 95% CI, 0.0%-1.5%) participants vs all other groups (3.5%; 95% CI, 2.9%-4.2%) (*P* = .008). Participants reporting that their family’s socioeconomic situation during adolescence was poor also had a higher prevalence of SMA (4.4%; 95% CI, 3.1%-5.7%) compared with all other groups (3.0%; 95% CI, 2.3%-3.7%) (*P* = .05). Furthermore, SMA prevalence was higher among sexual/gender minority individuals (5.8%; 95% CI, 3.7%-7.9%) compared with heterosexual or cisgender (2.9%; 95% CI, 2.3%-3.5%) participants (*P* = .01) (Figure). In subgroup analyses examining changes from 2021 to 2023 in SMA attempts, there was a significant increase among non-Hispanic White (1.6% to 2.7%; *P* = .01), US born (2.1% to 3.1%; *P* = .03), and sexual and gender minority (3.1% to 5.6%; *P* = .03) participants (Table 3).

**Figure.**
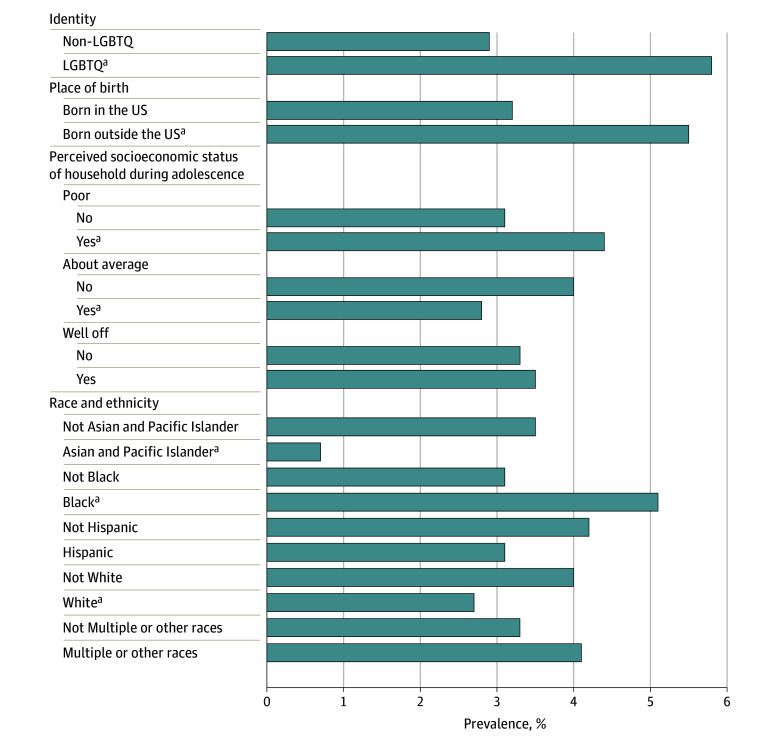
Adjusted and Weighted Estimates of the Prevalence of Attempts to Self-Manage Abortion (SMA) by Sociodemographic Characteristics, 2023 Findings shown for 7028 participants. Values represent estimated proportion who ever attempted SMA from a weighted logistic regression model, with SMA as the dependent variable and race and ethnicity; place of birth; lesbian, gay, transgender, queer, or nonbinary/gender nonconforming identity (LGBTQ); and perceived socioeconomic status of household during adolescence as independent variables. Except for the categories Not Hispanic and Hispanic, all participants in the race and ethnicity categories were considered non-Hispanic. ^a^Significant at *P* < .05 from a binary contrast comparing everyone in a given subgroup with everyone not in that subgroup.

**Table 3.** Change in Weighted and Adjusted Prevalence of SMA Attempts Within Subpopulations, 2021-2023

Variable	Subpopulation, No.	Weighted % (95% CI)		Absolute difference, 2021-2023, % (95% CI)	*P* value^a^
		December 2021-January 2022	June-July 2023		
Financial situation during adolescence					
Well off	2354	2.5 (1.2 to 3.8)	3.6 (1.8 to 5.4)	1.1 (−1.1 to 3.3)	.30
About average	8342	2.0 (1.4 to 2.7)	2.8 (2.1 to 3.5)	0.8 (−0.1 to 1.7)	.08
Poor	3369	3.4 (2.1 to 4.7)	4.2 (2.9 to 5.6)	0.8 (−0.9 to 2.6)	.40
Race and ethnicity					
Hispanic	2825	3.9 (2.2 to 5.6)	4.0 (2.5 to 5.5)	0.1 (−1.8 to 2.1)	.89
Non-Hispanic Asian or Pacific Islander	521	0.9 (0.2 to 1.7)	0.8 (0.01 to 1.7)	−0.1 (−1.2 to 0.1)	.77
Non-Hispanic Black	1200	3.6 (1.8 to 5.4))	5.0 (2.7 to 7.2)	1.4 (−1.4 to 4.1)	.34
Non-Hispanic Multiple or Other	534	2.8 (0.1 to 5.4)	4.0 (1.1 to 6.9)	1.2 (−2.7 to 5.2)	.55
Non-Hispanic White	9060	1.6 (1.1 to 2.1)	2.7 (2.0 to 3.5)	1.1 (0.3 to 2.0)	.01
Nativity					
Born outside the US	1843	3.9 (2.2 to 5.6)	4.7 (2.8 to 6.6)	0.8 (−1.2 to 2.9)	.44
Born in the US	12 297	2.2 (1.6 to 2.7)	3.0 (2.4 to 3.7)	0.8 (0.06 to 1.7)	.03
LGBTQ identity					
No	12 127	2.3 (1.7 to 2.9)	2.9 (2.3 to 3.5)	0.6 (−0.2 to 1.4)	.16
Yes	2002	3.1 (1.5. 4.7)	5.6 (3.6 to 7.7)	2.5 (0.4 to 4.7)	.03

Abbreviations: LGBTQ, lesbian, gay, bisexual, transgender, queer, or nonbinary/gender nonconforming; SMA, self-managed abortion.

^a^ *P* value highlights difference in prevalence of SMA by year of the survey within that subpopulation from a weighted logistic regression model, with year as the independent variable.

Among individuals who reported an SMA attempt, the mean (SD) age at the first attempt was 20.7 (6.2) years in 2021 and 21.2 (5.9) years in 2023. Across both years, approximately 4 in 10 (45.3% in 2021 and 39.0% in 2023) reported that their first SMA attempt occurred before age 20 years. Approximately 1 in 5 (18.5% in 2021 and 20.8% in 2023) reported more than 1 SMA attempt in their lifetime.

The most common methods for SMA included herbs (29.8% in 2021 and 25.9% in 2023), emergency contraception before confirming the pregnancy (with another method) (28.6% in 2021 and 29.7% in 2023), hitting themselves in the stomach (22.7% in 2021 and 21.6% in 2023), and alcohol or other substances (17.9% in 2021 and 18.6% in 2023). Fewer reported using misoprostol (13.7% in 2021 and 15.7% in 2023) and mifepristone (6.6% in 2021 and 11.0% in 2023). One-half (52.4% in 2021 and 48.4% in 2023) of the participants reported using more than 1 method. One-third (37.5% in 2021 and 37.7% in 2023) used a pregnancy test with a positive result before their SMA attempt. Nearly 1 in 5 (18.5% in 2021 and 14.9% in 2023) reported experiencing a complication requiring treatment by a physician or nurse with fewer (7.1% in 2021 and 4.7% in 2023) reporting seeking care at a hospital, emergency department, or urgent care. The complications most often reported were bleeding (8.3% in 2021 and 5.5% in 2023) and pain (8.3% in 2021 and 5.5% in 2023).

The most frequently cited reasons for SMA included being early in the pregnancy (33.3% in 2021 and 31.8% in 2023) and privacy (30.4% in 2021 and 32.2% in 2023). Almost 1 in 5 participants reported that the clinic was too expensive (16.7% in 2021 and 18.2% in 2023) or they preferred trying something on their own first before going to a clinic (20.8% in 2021 and 19.1% in 2023). Some cited concerns about encountering protestors at a clinic (13.7% in 2021 and 12.7% in 2023) or needing a parent’s consent (11.3% in 2021 and 8.9% in 2023) (Table 4).

**Table 4.** Details of Participants’ Experience With SMA Attempts by Year

Variable	No. (unweighted %)	
	December 2021-January 2022 (n = 168)	June-July 2023 (n = 236)
Age at first or only SMA, y^a^		
≤17	48 (28.6)	54 (22.9)
18-19	28 (16.7)	38 (16.1)
20-24	37 (22.0)	58 (24.6)
25-29	19 (11.3)	26 (11.0)
30-34	11 (6.6)	12 (5.1)
35-39	2 (1.2)	8 (3.4)
≥40	2 (1.2)	1 (0.4)
Refused	21 (12.5)	39 (16.5)
Decade when most recent or only SMA took place		
1970s or 80s	6 (3.6)	0
1990s	21 (12.5)	21 (8.9)
2000s	55 (32.7)	62 (26.3)
2010s	55 (32.7)	82 (34.8)
2020s	7 (4.2)	30 (12.7)
Missing	24 (14.3)	41 (17.4)
>1 SMA attempt in lifetime	31 (18.5)	49 (20.8)
Region lived in at time of SMA attempt		
New England (Connecticut, Maine, Massachusetts, New Hampshire, Rhode Island, Vermont)	5 (3.0)	4 (1.7)
Mid-Atlantic (New Jersey, New York, Pennsylvania)	18 (10.7)	26 (11.0)
East North Central (Illinois, Indiana, Michigan, Ohio, Wisconsin)	27 (16.1)	38 (16.1)
West North Central (Iowa, Kansas, Minnesota, Missouri, Nebraska, North Dakota, South Dakota)	7 (4.2)	17 (7.2)
South Atlantic (Delaware, Florida, Georgia, Maryland, North Carolina, South Carolina, Virginia, District of Columbia, West Virgina)	29 (17.3)	31 (13.1)
East South Central (Alabama, Kentucky, Mississippi, Tennessee)	4 (2.4)	11 (4.7)
West South Central (Arkansas, Louisiana, Oklahoma, Texas)	19 (11.3)	26 (11.0)
Mountain (Arizona, Colorado, Idaho, Montana, Nevada, New Mexico, Utah, Wyoming)	14 (8.3)	13 (5.5)
Pacific (Alaska, California, Hawaii, Oregon, Washington)	17 (10.1)	39 (16.5)
Missing	28 (16.7)	31 (13.1)
Method use^b^		
Herbs	50 (29.8)	61 (25.9)
EC before confirming pregnancy^c^	48 (28.6)	70 (29.7)
Hit in the stomach	39 (22.7)	51 (21.6)
Taking a hot bath or shower	33 (19.6)	27 (11.4)
Misoprostol and/or mifepristone	31 (18.0)	58 (24.1)
Misoprostol	23 (13.7)	37 (15.7)
Mifepristone	11 (6.6)	26 (11.0)
Alcohol or other substances	30 (17.9)	44 (18.6)
Lifting heavy objects	26 (15.5)	21 (8.9)
EC after confirming pregnancy	21 (12.5)	32 (13.6)
Some other drug or medication	15 (8.9)	31 (13.1)
Something else	10 (5.9)	8 (3.4)
Inserting an object in body	6 (3.6)	9 (3.8)
Pregnancy test before SMA attempt		
No test, do not remember, or refused	96 (57.1)	129 (55.9)
Took a test and it was positive	63 (37.5)	87 (37.7)
Took a test and it was negative or unclear	9 (5.4)	15 (6.5)
Outcome		
Methods worked to end the pregnancy	56 (33.3)	77 (32.6)
Abortion at a clinic	32 (19.1)	46 (19.5)
Continued the pregnancy	23 (13.7)	32 (13.6)
Had a miscarriage later in the pregnancy	8 (4.8)	17 (7.2)
Nothing/maybe never pregnant	40 (23.8)	56 (23.7)
Missing	9 (5.4)	8 (3.4)
Experienced a complication requiring treatment by a physician or nurse		
Yes	31 (18.5)	35 (14.9)
No	131 (78.0)	191 (80.9)
Missing	6 (3.5)	10 (4.2)
Type of complication^b^		
Bleeding	14 (8.3)	13 (5.5)
Pain	14 (8.3)	13 (5.5)
Fever	7 (4.2)	2 (0.9)
Nausea^d^	NA	9 (3.8)
Vomiting^d^	NA	1 (0.4)
Diarrhea^d^	NA	2 (0.8)
Something else	3 (1.8)	5 (2.1)
Sought care at hospital, ED, or urgent care		
Yes	12 (7.1)	11 (4.7)
No	156 (92.9)	225 (95.3)
Reasons for SMA^b^		
I was early in the pregnancy	56 (33.3)	75 (31.8)
It seemed more private	51 (30.4)	76 (32.2)
I preferred trying something on my own first	35 (20.8)	45 (19.1)
Doing on my own seemed easier or faster	29 (17.3)	37 (15.7)
It felt like less of an abortion	29 (17.3)	26 (11.0)
Clinic was too expensive	28 (16.7)	43 (18.2)
I wanted to avoid protestors	23 (13.7)	30 (12.7)
I thought I needed my parent’s consent	19 (11.3)	21 (8.9)
Didn’t know where clinic was that could help	18 (10.7)	22 (9.3)
Doing it on my own seemed more natural	16 (9.5)	21 (8.9)
Clinic was too far away or hard to get to	14 (8.3)	15 (6.4)
I use vitamins or herbs whenever I am sick	14 (8.3)	15 (6.4)
Abortion is illegal^d^	NA	14 (5.9)

Abbreviations: EC, emergency contraception; ED, emergency department; SMA self-managed abortion.

^a^ If more than 1 SMA attempt was reported, age at first attempt is used here.

^b^ Proportions do not sum to 100% as participants could select multiple responses and write-in responses.

^c^ These participants also reported using another method for SMA in addition to EC before confirming pregnancy; those who reported EC before confirming pregnancy as their only method were excluded from all estimates.

^d^ Additional response option offered only in the 2023 survey.

## Discussion

To our knowledge, this study represents the first population-based estimate of changes in attempts to self-manage abortion before and after the *Dobbs* decision. We observed an increase in the proportion of the US female population of reproductive age that reported experience with SMA from 2.4% in 2021 to 3.4% in 2023, suggesting people are increasingly relying on self-sourced methods to end a pregnancy. This is likely a conservative estimate, given underreporting of abortion in self-administered surveys. Assuming people underreport SMA to the same degree they do past-year, facility-based abortion, the proportion with SMA experience increased from approximately 5% before *Dobbs* to 7% after *Dobbs*.

These data offer the opportunity to disentangle the relative frequency of SMA attempts in confirmed vs suspected pregnancies, both of which we hypothesize might increase and are important to measure after *Dobbs*. As barriers to facility-based abortion grow, SMA may increasingly become an individual’s only or preferred option to end a pregnancy. However, additionally, as people fear criminalization for seeking pregnancy-related care or worry about the accessibility of abortion, there may be increased reliance on proactive efforts to remain nonpregnant, regardless of confirmation of the pregnancy with a medical test. This practice, sometimes referred to as menstrual regulation, is already well documented globally where abortion is not legally available.^25^ Given the availability of and interest in taking mifepristone and/or misoprostol in the context of a late period,^26,27^ it would not be surprising to see an increase in preventive efforts after *Dobbs*.

An advantage of this large population-based study is its ability to identify differences in the use of SMA by sociodemographic characteristics. We found that experience with SMA was higher among socially oppressed groups, including racial and ethnic minoritized individuals and people identifying with sexual and gender minoritized groups. Although not an unexpected finding, given that these groups also report more barriers to reproductive health care, medical mistreatment, and foregone health care,^28,29,30,31^ this may serve as a reminder of who may need additional support accessing safe abortion care moving forward.

Consistent with earlier research,^7,9,12,32^ participants used a wide range of methods beyond self-sourced mifepristone and/or misoprostol for SMA, including several with the potential for harm and many that likely offer low to no effectiveness in ending a pregnancy. Thus, interaction with the health care system following SMA is not uncommon, whether to seek emergency care related to adverse effects or complications or to seek subsequent prenatal or abortion care. It is therefore critical that clinicians across medical specialties, but particularly those in emergency and primary care settings, be aware of potential complications associated with SMA as well as its usual clinical course.^33^ Furthermore, given recent evidence that clinicians are the most common way in which people with SMA are connected with the carceral system, clinicians must ensure that they protect patient privacy, especially in settings where abortion is increasingly criminalized.^34,35^

### Limitations

There were limitations to this study. Most importantly, we asked participants to report on a sensitive, stigmatized, and now, in some settings, criminalized behavior. While we anticipate some consistent underreporting over time, we remain concerned about differential misreporting of SMA from 2021 to 2023, which would introduce bias that our pre-post design is unable to overcome. The direction of this potential misreporting is unclear. While increased criminalization of pregnancy and abortion after *Dobbs* could make someone less likely to disclose their prior SMA experience, with increased public attention and conversation around abortion rights, people might be more emboldened to disclose their own SMA experience. Furthermore, the recent expansion in virtual and telehealth medication abortion may have introduced some confusion to participants in the definition of SMA. To ensure reliability of our estimates, we used consistent language across surveys that defines SMA as something taking place without medical assistance. However, this definition may need clarification moving forward. Finally, SMA is a relatively rare occurrence. While our sample size is powered to detect changes in SMA from 2021 to 2023, it is likely underpowered to detect changes within subgroups.

## Conclusions

In this rigorous pre-post population-based survey, we found evidence of increased SMA attempts from before to after the Supreme Court’s decision overturning federal protections on abortion. The national landscape of abortion access will likely continue to become more restricted or at least remain in flux, for years to come, suggesting SMA attempts will continue to increase. Efforts to connect people who are pregnant with safe and effective methods of SMA with medication abortion pills, as well as efforts to ensure health care clinicians are aware of SMA, may help mitigate some of the legal and health risks people who attempt SMA will face.

## Notes

Supplement 1. **eTable.** Comparing the Sociodemographic Profile of the Study Samples to the Census and the National Survey of Family Growth **eMethods.** Calculation of the Multiplier to Account for Underreporting of Abortion

Supplement 2.
Data Sharing Statement
