# Supplementary material for: Self-Managed Abortion Attempts Before vs After Changes in Federal Abortion Protections in the US
Source: JAMA Netw Open. 2024 Jul 30;7(7):e2424310. doi: 10.1001/jamanetworkopen.2024.24310 (PMC12312524; doi:10.1001/jamanetworkopen.2024.24310)
Supplement: Supplement 1. — eTable. Comparing the Sociodemographic Profile of the Study Samples to the Census and the National Survey of Family Growth eMethods. Calculation of the Multiplier to Account for Underreporting of Abortion [file jamanetwopen-e2424310-s001.pdf]

Supplementary Online Content

Ralph L, Schroeder R, Kaller S, Grossman D, Biggs MA. Self-managed abortion attempts before vs after changes in federal abortion protections in the US. *JAMA Netw Open*. 2024;7(7):e2424310. doi:10.1001/jamanetworkopen.2024.24310

eTable. Comparing the Sociodemographic Profile of the Study Samples to the Census and the National Survey of Family Growth

eMethods. Calculation of the Multiplier to Account for Underreporting of Abortion

This supplementary material has been provided by the authors to give readers additional information about their work.

© 2024 Ralph L et al. *JAMA Network Open.*

eTable. Comparing the Sociodemographic Profile of the Study Samples to Census and the National Survey of Family Growth

	Census/American Community Survey Estimates %	National Survey of Family Grown, 2017-2019 (N=5557) Weighted %[a]	Study Sample, 2021 (N=6841) Weighted %[a]	Study Sample, 2023 (N=6785) Weighted %[a]
Age (years)				
15 to 19	13.7[a]		11.5	14.5
20 to 24	14.1		13.1	13.3
25 to 29	15.0		15.6	14.5
30 to 34	15.3		16.0	15.4
35 to 39	14.7		15.3	14.8
40 to 44	13.9		14.5	14.3
45 to 49	13.3		14.0	13.2
Household percent of Federal Poverty Level[b]				
<100%	13.0		12.0	10.0
≥100%	87.0		88.0	90.0
Race and ethnicity (limited to age ≤ 44) [c]				
Hispanic	21.0		20.9	21.2
Non-Hispanic Black	14.6		13.9	13.9
Non-Hispanic Other	8.5		10.0	10.5
Non-Hispanic White	55.9		55.1	54.4
Highest level of education completed (limited to age > 17 years) [d]				
< High school	8.4		8.2	8.3
High school diploma or GED	22.9		21.6	24.1
Some college or associate's degree	29.5		29.3	28.2
College degree	39.3		40.9	39.5
Ever been pregnant				
Yes		65.8	57.9	54.3
No		34.2	41.5	45.3
Refused		n/a	0.6	0.4

Notes: [a] Survey weights applied; [b] Age distribution accessed from https://www.census.gov/data/tables/2021/demo/age-and-sex/2021-age-sex-composition.html (Table 1); [c] Bridged-Race Population Estimates (2020) accessed from CDC Wonder Online Database: http://wonder.cdc.gov/bridged-race-v2020.html [d] Educational attainment (2021) accessed from https://www.census.gov/data/tables/2021/demo/age-and-sex/2021-age-sex-composition.html (Table 3).

© 2024 Ralph L et al. *JAMA Network Open.*

eMethods. Calculation of the multiplier to account for underreporting of abortion.

Data sources:

Measure	Data Source	Notes
Proportion of the U.S. population reporting a past year abortion		
Numerator: # of facility-based abortions provided in 2020	Guttmacher Abortion Provider Census [21]	Restricted to age ≥ 18 years
Denominator: # of reproductive age women living in the U.S. in 2020	U.S. Census [22]	Restricted to ages 18 to 49
Proportion of survey respondents reporting a past year abortion	2021/2022 survey	Restricted to age ≥ 18 years

Notes:

Calculations:

National estimate of the percent of U.S. population reporting a past year abortion (2020) = 896,674 / 67,996,000 = 1.32%

Survey estimate of the weighted percent of respondents (2021/22) that reported a past year abortion = 0.62% (weighted %)

Multiplier = National estimate / Survey estimate = 1.32/0.62 = 2.1

Additional notes:

The Guttmacher Abortion Provider Census is generally considered to be the most complete estimate of facility-based abortion in the U.S., and the last national estimate from prior to the Dobbs decision and closure of many abortion facilities. Thus, we use only 2021/22 data to develop the multiplier.

© 2024 Ralph L et al. *JAMA Network Open.*
