# Supplementary material for: Self-Managed Abortion Attempts Before vs After Changes in Federal Abortion Protections in the US
Source: JAMA Netw Open. 2024 Jul 30;7(7):e2424310. doi: 10.1001/jamanetworkopen.2024.24310 (PMC12312524; doi:10.1001/jamanetworkopen.2024.24310)
Supplement: Supplement 2. — Data Sharing Statement [file jamanetwopen-e2424310-s002.pdf]

# Data Sharing Statement

Ralph. Self-Managed Abortion Attempts Before vs After Changes in Federal Abortion Protections in the US. *JAMA Netw Open*. Published July 30, 2024.
doi:10.1001/jamanetworkopen.2024.24310

**Data**
**Data available:** No

**Additional Information**
**Explanation for why data not available:** A limited version of the dataset can be made available upon reasonable request to the Corresponding Author.
